# A Novel Flavonoid Glucoside from the Fruits of *Lycium ruthenicun*

**DOI:** 10.3390/molecules23020325

**Published:** 2018-02-03

**Authors:** Jing-Jing Qi, Yong-Ming Yan, Li-Zhi Cheng, Bao-Hua Liu, Fu-Ying Qin, Yong-Xian Cheng

**Affiliations:** 1 School of Pharmacy, Yunnan University of Traditional Chinese Medicine, Kunming 650500, China; qijing023@163.com; 2 State Key Laboratory of Phytochemistry and Plant Resources in West China, Kunming Institute of Botany, Chinese Academy of Sciences, Kunming 650201, China; qinfuying@mail.kib.ac.cn; 3 Guangdong Key Laboratory for Genome Stability & Disease Prevention, School of Pharmaceutical Sciences, School of Medicine, Shenzhen University Health Science Center, Shenzhen 518060, China; yanym@szu.edu.cn (Y.-M.Y.); 13424039397@163.com (L.-Z.C.); 4 School of Pharmacy, Henan University of Chinese Medicine, Zhengzhou 450008, China

**Keywords:** *Lycium ruthenicun*, flavonoid, ruthenicunoid A, SIRT1

## Abstract

A novel flavonoid glucoside, ruthenicunoid A (**1**), together with eight known substances, were isolated from the fruits of *Lycium ruthenicun* Murr. Their structures were elucidated by extensive spectroscopic data and chemical methods. Especially, the absolute configuration of glucose residue in **1** was assigned by acid hydrolysis followed by derivatization and GC analysis. Biological evaluation towards Sirtuin 1 (SIRT1) found that compounds **1** and **2** exhibit inhibitory activity against SIRT1 in a concentration-dependent manner, indicating its potential on SIRT1-associated disorders.

## 1. Introduction

*Lycium ruthenicun* Murr. is found in the northwest regions of China. Its fruit is edible and has been used as a remedy for the treatment of hypertension, ureteral stones, tinea and furuncle, and gingvial bleeding [1,2,3]. The fruits of *L. ruthenicun* contains a variety of bioactive ingredients, in particular, polyphenols such as anthocyanins, which have antioxidant effects and are beneficial for the prevention and treatment of cardiovascular diseases are rich in the fruits [4,5]. A literature search found that the major research in the past focused on the extraction methods and measurement of the total anthocyanins [6,7,8]; no comprehensive study has been conducted to explore the chemical constituents of *L. ruthenicun.* This attracted our attention. In the course of continuous study, a new flavonoid glucoside, ruthenicunoid A, and eight known compounds were isolated and identified. All the compounds were tested for their biological activity on SIRT1, a nicotinamide adenosine dinucleotide (NAD)-dependent deacetylase. Our efforts will be described below.

## 2. Results and Discussion

### 2.1. Structure Elucidation of the Compounds

**Figure 1:**
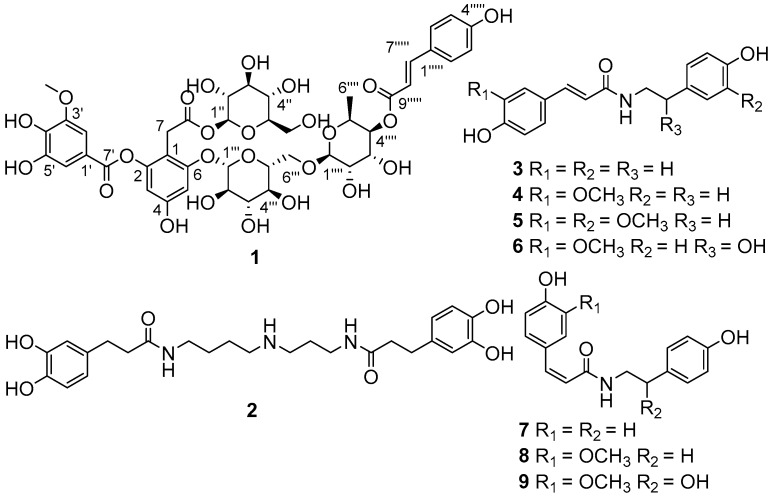
Chemical structures of compounds **1**–**9**.

The EtOH extract of *L. ruthenicun* was suspended in water and partitioned with EtOAc. The EtOAc soluble part was submitted to a combination of chromatography to afford compounds **1***–***9** (Figure 1).

**Figure 2:**
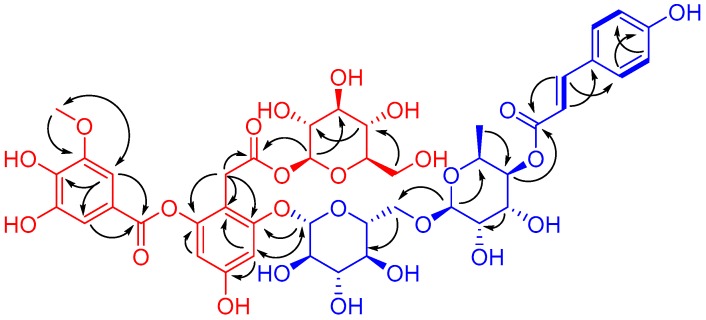
^1^H-^1^H COSY (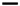) and key HMBC (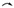) and ROESY (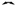) correlations of **1**.

Compound **1**, obtained as a brownish auburn gum, has the molecular formula C_43_H_50_O_25_ (19 degrees of unsaturation) based on analysis of its HRESIMS at *m*/*z* 989.2546 [M + Na]^+^ (calcd. for C_43_H_50_O_25_Na, 989.2539). The ^1^H NMR spectrum of 1 (Table 1) shows an AABB coupling system characteristic of a group of protons at *δ*_H_ 7.48 (2H, d, *J* = 8.5 Hz, H-2′′′′′, 6′′′′′) and 6.81 (2H, d, *J* = 8.5 Hz, H-3′′′′′, 5′′′′′), four aromatic protons at *δ*_H_ 6.42 (1H, d, *J* = 1.8 Hz, H-3), *δ*_H_ 6.67 (1H, d, *J* = 1.8 Hz, H-5), *δ*_H_ 7.30 (1H, d, *J* = 1.8 Hz, H-2′), and *δ*_H_ 7.35 (1H, d, *J* = 1.8 Hz, H-6′), suggesting the presence of two 1,2,3,5-tetrasubstituted benzene rings. In addition, one methoxy group at *δ*_H_ 3.88 (3H, s, 3′-OCH_3_) and two olefinic protons respectively at *δ*_H_ 7.63 (1H, d, *J* = 15.9 Hz, H-7′′′′′) and *δ*_H_ 6.37 (1H, d, *J* = 16.0 Hz, H-8′′′′′) were observed. The ^13^C NMR and DEPT spectra of 1 (Table 1) show 43 carbon signals attributed to two methyl (one oxygenated), three sp^3^ methylene, twenty-five methine (ten olefinic and fifteen aliphatic), and thirteen quaternary carbons (three carbonyls, ten sp^2^ including seven oxygenated). Inspection of these NMR data found that the partial signals resemble those of malvone [9,10], differing in that 5′-OMe in malvone was replaced by 5′-OH in 1. The HMBC correlation (Figure 2) of OCH_3_/C-3′ and ROESY correlation of OCH_3_/H-2′ (Figure 2), in consideration of the chemical shifts of C-4′ (*δ*_C_ 141.6), C-5′ (*δ*_C_ 146.5), secured the presence of 3-methoxy,4,5-dihydroxyl substituted pattern. Further HMBC correlations of H-1′′/C-8, H-1′′′/C-6, H-7/C-1, C-2, C-6, in consideration of chemical shifts of C-2, C-4, and C-6 indicated the position of two glucose residues. HMBC correlations of H-2′, H-6′/C-7′ and the significant upfield shift of C-2 (*δ*_C_ 152.1) secured an ester carbonyl attached to C-2 instead of C-4. Apart from the red part, the remaining signals (blue part) are in accordance with those of 4-*p*-cumaroyl-α-rhamnosyl-(1 → 6)-β-glucose [11]. The observation of the above-mentioned AABB coupling system, a transformed double bond (*J*_H-7′′′′′,H-8′′′′′_ = 15.9 Hz), and two sugar moieties in the middle field supported our conclusion. Additional HMBC cross peaks of H-1′′′′/C-6′′′, H-4′′′′/C-9′′′′′ further indicated the linkage pattern in the blue part of 1. The red and blue parts were connected via C-6-O-C-1′′′ supported by the HMBC correlation of H-1′′′/C-6 and the ROESY correlation of H-5/H-1′′′. Thus, the planar structure of 1 was deduced. For the configuration of the sugar moieties, acid hydrolysis of 1 followed by TLC comparison and GC analysis allowed the assignment of d-glucose and l-rhamnose. In detail, the L-cysteine methyl ester hydrochloride derivatives of the hydrolysis product of 1, d-, l-glucose and L-rhamnose were prepared and subjected to GC analysis. The retention time for that of 1 is 17.698 min and 21.290 min, close to that of L-rhamnose (17.847 min) and d-glucose (21.276 min) rather than l-glucose (21.768 min), clarifying the type of sugar and its configuration. It should be noted that d-rhamnose or d,l-rhamnose in this study was not readily available, so that the derivative of d-rhamnose couldn’t be prepared and analyzed by GC. However, it is possible to differentiate l- from d-form of rhamnose by comparing the consistency of retention time between the derivative of l-rhamnose and that of the mixture of l-rhamnose with 1. In this way, we found that the retention time for l-cysteine methyl ester hydrochloride derivative of l-rhamnose is identical with that of co-injection of the mixture (16.827 min for the latter) by GC/MS analysis, securing the type of rhamnose and its configuration accordingly. Taken together, the structure of 1 was identified and named as ruthenicunoid A.

By analysis of the NMR spectroscopic data and comparison with the literature, the known compounds were respectively identified as *N^1^*,*N^10^*-bis(dihydrocaffeoyl)spermidine (**2**) [12], *N*-*trans*-coumaroyltyramine (**3**) [13], *N*-*trans*-feruloyltyramine (**4**) [14], *N*-*trans*-feruloyl 3′-O-methyldopamine (**5**) [15], *N*-*trans*-feruloyloctopamine (**6**) [14], *N*-*cis*-coumaroyltyramine (**7**) [16], *N*-*cis*-feruloyltyramine (**8**) [14], and *N*-*cis*-feruloyloctopamine (**9**) [14].

### 2.2. Biological Evaluation

**Figure 3:**
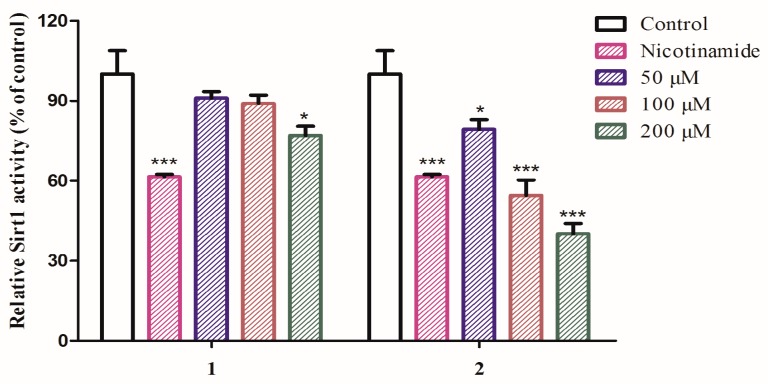
SIRT1 activation of compounds **1** and **2**. SIRT1 enzyme activity was measured using the SIRT1 Fluorometric Drug Discovery Kit. Statistical analysis was performed using one-way analysis of the variance (ANOVA) followed by Bonferroni’s multiple comparison tests. All error bars are S.E.M. * *p* < 0.05, *** *p* < 0.001 versus control (*n* = 3).

SIRT1 is a nicotinamide adenosine dinucleotide (NAD)-dependent deacetylase which regulates a wide range of cellular functions and is implicated in many diseases such as aging, cancer and so on [17,18,19,20]. So far, several SIRT1 activators and inhibitors such as nicotinamide (IC_50_ value less than 50 μM), salermide (IC_50_ value = 76.2 μM), and cambinol (IC_50_ value = 56 μM) were documented [21]. With this assay in hand and considering the title species is used for aging, compounds **1**–**9** were thus tested for their inhibitory activity against SIRT1. The results showed that compounds **1** and **2** are active towards SIRT1 (Figure 3) with **2** to be more potent than **1**, comparable to that of nicotinamide at the concentration of 100 μM, whereas compounds **3**–**9** are not active (data not shown). The finding of **2** as a SIRT1 inhibitory substance indicated that such type of amide or aliphatic amine might be of important structure class for antiaging drug design.

## 3. Experimental Section

### 3.1. General Procedures

Optical rotations were recorded on a Horiba SEPA-300 polarimeter. UV spectrum was recorded on a Shimadzu UV-2401PC spectrometer (Shimadzu Corporation, Tokyo, Japan). GC analysis was performed using an Agilent 6890N gas chromatography instrument (Agilent Technologies, Santa Clara, CA, USA). GC/MS analysis was performed using an Agilent 7890B GC System (Agilent Technologies, Santa Clara, CA, USA) and a Asilent 5977 MSD inrun (Agilent Technologies, Santa Clara, CA, USA). NMR spectra were recorded on a Bruker AV-400 (Bruker, Karlsruhe, Germany) or an AV-600 spectrometer (Bruker, Karlsruhe, Germany), with TMS as an internal standard. ESIMS, and HRESIMS were measured on an Agilent G6230TOF MS spectrometer (Agilent Technologies, Santa Clara, CA, USA). C-18 silica gel (40–60 μm; Daiso Co., Tokyo, Japan), MCI gel CHP 20P (75–150 μm, Mitsubishi Chemical Industries, Tokyo, Japan) and Sephadex LH-20 (Amersham Pharmacia, Uppsala, Sweden) were used for column chromatography. Semi-preparative HPLC was carried out using an Agilent 1200 liquid chromatograph with a YMC-Pack ODS-A column (250 mm × 10 mm, i.d., 5 μm) and Thermo Hypersil GOLD-C_18_ column (250 mm × 21.2 mm, i.d., 5 μm).

### 3.2. Plant Material

The fruits of *L. ruthenicum* were collected from the market of herbal medicine in Yunnan province, People’s Republic of China, in September 2016. The material was identified by Mr. Bin Qiu at Yunnan Institute of Materia Medica, and a voucher specimen (CHYX-0605) is deposited at the State Key Laboratory of Phytochemistry and Plant Resources in West China, Kunming Institute of Botany, Chinese Academy of Sciences, People’s Republic of China.

### 3.3. Extraction and Isolation

The fruits of *L. ruthenicum* (5 kg) were powdered and soaked by 80% aqueous EtOH (3 × 25 L × 24 h) to give a crude extract, which was suspended in water followed by extraction with EtOAc to afford an EtOAc soluble extract (85 g). The EtOAc extract was divided into six parts (Fr.1–Fr.6) by using a MCI gel CHP 20P column eluted with gradient aqueous MeOH (20–100%). Fr.2 (3.5 g) was purified by Sephadex LH-20 (MeOH) followed by semipreparative HPLC (MeOH/H_2_O, 27:73, containing 0.05% formic acid) to afford compound **2** (78.4 mg, *t*_R_ = 9.8 min). Fr.4 (10.1 g) was separated by Sephadex LH-20 (MeOH) to yield six fractions (Fr.4.1–Fr.4.6). Fr.4.3 (2.1 g) was separated by RP-18 column (MeOH/H_2_O, 30–100%) to get three fractions (Fr.4.3.1–Fr.4.3.3). Fr.4.3.3 (490 mg) was separated by Sephadex LH-20 (MeOH) to yield four fractions (Fr.4.3.3.1–Fr.4.3.3.4). Among these, Fr.4.3.3.4 (48 mg) was purified by semi-preparative HPLC (MeCN/H_2_O, 28:72) to yield compounds **4** (2.1 mg, *t*_R_ = 16.1 min) and **5** (2.3 mg, *t*_R_ = 21.3 min). Fr.4.4 (1.0 g) was separated by RP-18 column (MeOH/H_2_O, 35–100%) to get five fractions (Fr.4.4.1–Fr.4.4.5). Fr.4.4.2 (180 mg) was separated by preparative HPLC (MeOH/H_2_O, 10–100%) to get three fractions (Fr.4.4.2.1–Fr.4.4.2.3). Fr.4.4.2.1 (23 mg) was purified by semi-preparative HPLC (MeCN/H_2_O, 21:79) to afford compound **1** (4.9 mg, *t*_R_ = 15.4 min). Fr.4.4.3 (380 mg) was separated by preparative HPLC (MeOH/H_2_O, 10–100%) to get nine fractions (Fr.4.4.3.1–Fr.4.4.3.9). Of which, Fr.4.4.3.3 (56.3 mg) was purified by semipreparative HPLC (MeCN/H_2_O, 18:82) to afford compounds **6** (5.4 mg, *t*_R_ = 27.9 min) and **9** (1.0 mg, *t*_R_ = 30.3 min). Fr.4.4.3.7 (23 mg) was purified by semipreparative HPLC (MeCN/H_2_O, 27:73) to yield compound **7** (2.3 mg, *t*_R_ = 22.8 min). Fr.4.4.3.8 (44 mg) was purified by semi-preparative HPLC (MeCN/H_2_O, 23:77) to afford compounds **3** (7.1 mg, *t*_R_ = 27.0 min) and **8** (2.1 mg, *t*_R_ = 29.6 min).

### 3.4. Compound Characterization Data

Ruthenicunoid A (**1**): Brownish auburn gum; ${\left[\alpha \right]}_{D}^{21}$: −23.5 (*c* 0.49, MeOH). UV (MeOH) *λ*_max_ (log *ε*): 203 (4.66), 313 (4.47) nm. ESIMS *m*/*z*: 989 [M + Na]^+^. HRESIMS *m*/*z*: 989.2546 [M + Na]^+^ (calcd. for C_43_H_50_O_25_Na, 989.2539); ^1^H- and ^13^C-NMR, see Table 1.

### 3.5. Acid Hydrolysis and Sugar Analysis

A solution of **1** (1.0 mg) in 1 N HCl was stirred at 70 °C for 5 h. After cooling, the mixtures were extracted with EtOAc. The aqueous layer was neutralized with 1 N NaOH and concentrated in vacuo, which was subsequently dissolved in anhydrous pyridine (2 mL). To these solutions L-cysteine methyl ester hydrochloride (2.0 mg) was added, and the mixtures were stirred at 60 °C for 1 h and concentrated in vacuo at 0 °C. Slow addition of 1-(trimethylsiyl) imidazole to the mixtures was followed by stirring at 60 °C for 2 h. Aliquots (4 µL) of the supernatants were subjected to chiral GC analysis to determine that D-glucose and L-rhamnose unitis are present in **1** [22,23].

### 3.6. SIRT1 Inhibition

For examination of SIRT1 inhibition of the compounds, each well contained 0.5 U (1 U = 1 pmol/min at 37 °C) of SIRT1 enzyme, 1000 μM of NAD^+^ (Enzo Life Sciences, Farmingdale, NY, USA), 100 μM of SIRT1 peptide substrate (Enzo Life Sciences) and SIRT1 assay buffer (50 mM Tris-HCl, pH 8.0, 137 mM NaCl, 2.7 mM KCl, 1 mM MgCl_2_, 1 mg/mL BSA) along with the test compounds at a concentration of 50, 100 and 200 μM, respectively. Nicotinamide, a known inhibitor of SIRT1 enzyme was used as a control at a concentration of 100 μM. The plate was incubated at 37 °C for 30 min and the reaction was stopped using Fluor de Lys developer II solution (Enzo Life Sciences) containing 2 mM nicotinamide. The plate was further incubated at 37 °C for another 30 min and the samples were read by a fluorimeter with an excitation wavelength of 360 nm and emission wavelength of 460 nm [24].

## 4. Conclusions

To conclude, this study led to the isolation of a new flavonoid glucoside and eight known amide derivatives from the edible fruits of *L. ruthenicun*. Biological evaluation found that both **1** and **2** showed inhibitory activity against SIRT1, indicating their roles in SIRT1-associated disorders and suggesting **2** to be a potent structure template worth for further optimization as SIRT1 inhibitors.

## Figures and Tables

**Table 1:** ^1^H (600 MHz) and ^13^C NMR (150 MHz) data of **1** (*δ* in ppm, *J* in Hz, methanol-*d*_4_).

	1				
No.	*δ*_H_	*δ*_C_	No.	*δ*_H_	*δ*_C_
1		109.2	1′′′	4.87, brs	103.2
2		152.1	2′′′	3.51, m	74.8
3	6.42, d, 1.8	105.2	3′′′	3.47, m	77.8
4		159.1	4′′′	3.32, overlap	71.0
5	6.67, d, 1.8	102.4	5′′′	3.32, overlap	77.7
6		158.6	6′′′	3.96, m	67.9
7	3.73, m	30.4		3.62, m	
	3.66, m		1′′′′	4.76, brs	102.2
8		172.2	2′′′′	3.43, m	78.2
1′		120.1	3′′′′	3.86, m	70.4
2′	7.30, d, 1.8	106.9	4′′′′	5.00, m	75.3
3′		149.3	5′′′′	3.79, m	67.9
4′		141.6	6′′′′	1.04, d, 6.2	17.8
5′		146.5	1′′′′′		127.2
6′	7.35, d, 1.8	112.8	2′′′′′	7.48, d, 8.5	131.3
7′		166.5	3′′′′′	6.81, d, 8.5	116.8
1′′	5.45, d, 8.2	96.0	4′′′′′		161.2
2′′	3.89, m	72.1	5′′′′′	6.81, d, 8.5	116.8
3′′	3.30, m	73.8	6′′′′′	7.48, d, 8.5	131.3
4′′	3.42, m	71.2	7′′′′′	7.63, d, 15.9	146.9
5′′	3.50, m	77.7	8′′′′′	6.37, d, 15.9	115.2
6′′	3.92, m	62.5	9′′′′′		169.1
	3.74, m		-OCH_3_	3.88, s	56.9

## Supplementary Materials

The following data are available online.
