# Supplementary material for: A Novel Flavonoid Glucoside from the Fruits of Lycium ruthenicun
Source: Molecules. 2018 Feb 3;23(2):325. doi: 10.3390/molecules23020325 (PMC6017501; doi:10.3390/molecules23020325)
Supplement: Supplementary file 1 [file molecules-23-00325-s001.pdf]

Supplementary Materials: A novel flavonoid glucoside from the fruits of *Lycium ruthenicun*

Jing-Jing Qi, Yong-Ming Yan, Li-Zhi Cheng, Bao-Hua Liu, Fu-Ying Qin, Yong-Xian Cheng

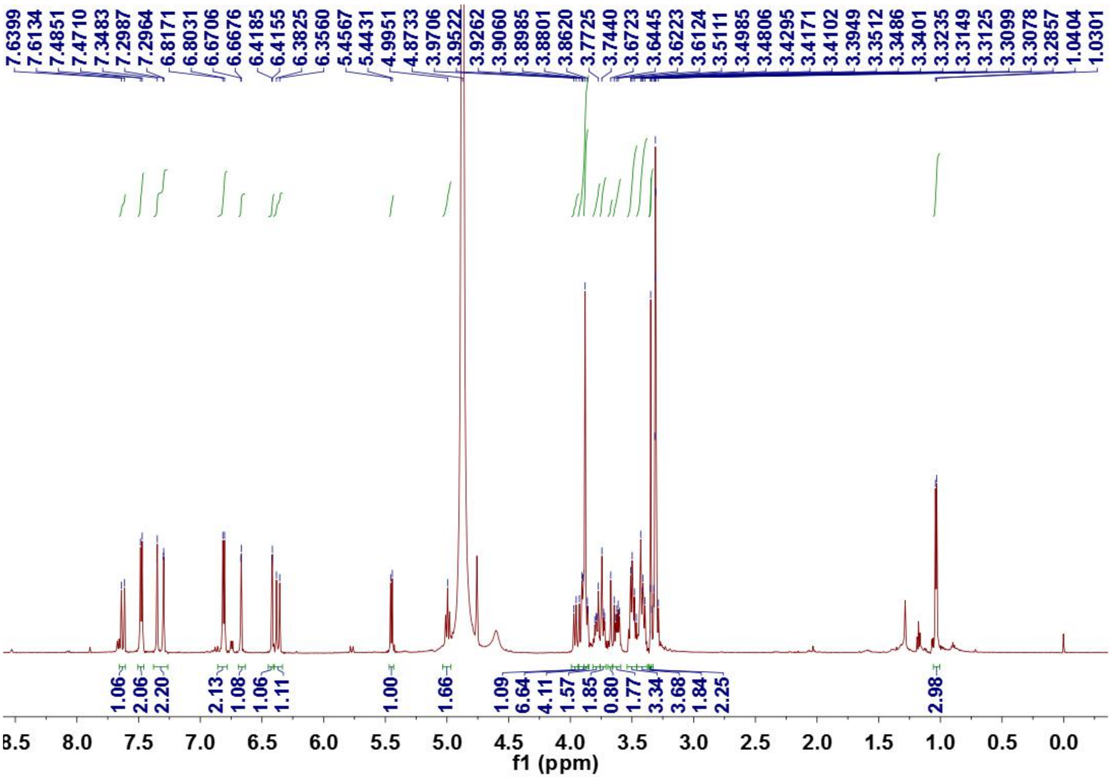

Figure S1. ^{1}H NMR spectrum of **1** in methanol-d_4

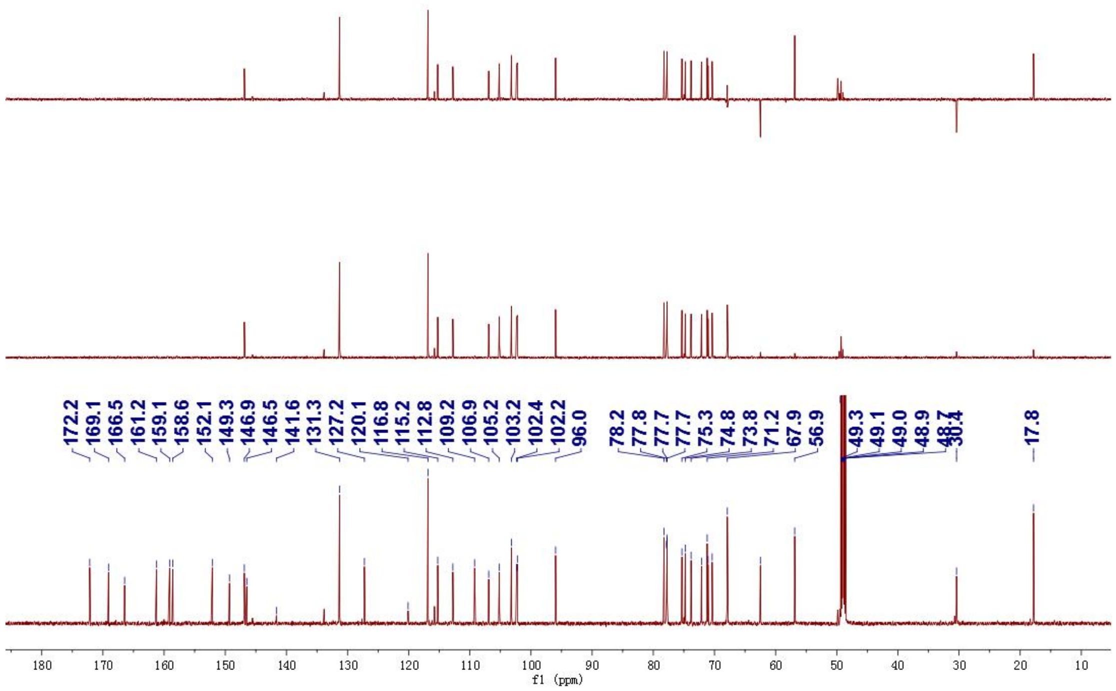

Figure S2. ^{13}C NMR and DEPT spectra of **1** in methanol-d_4

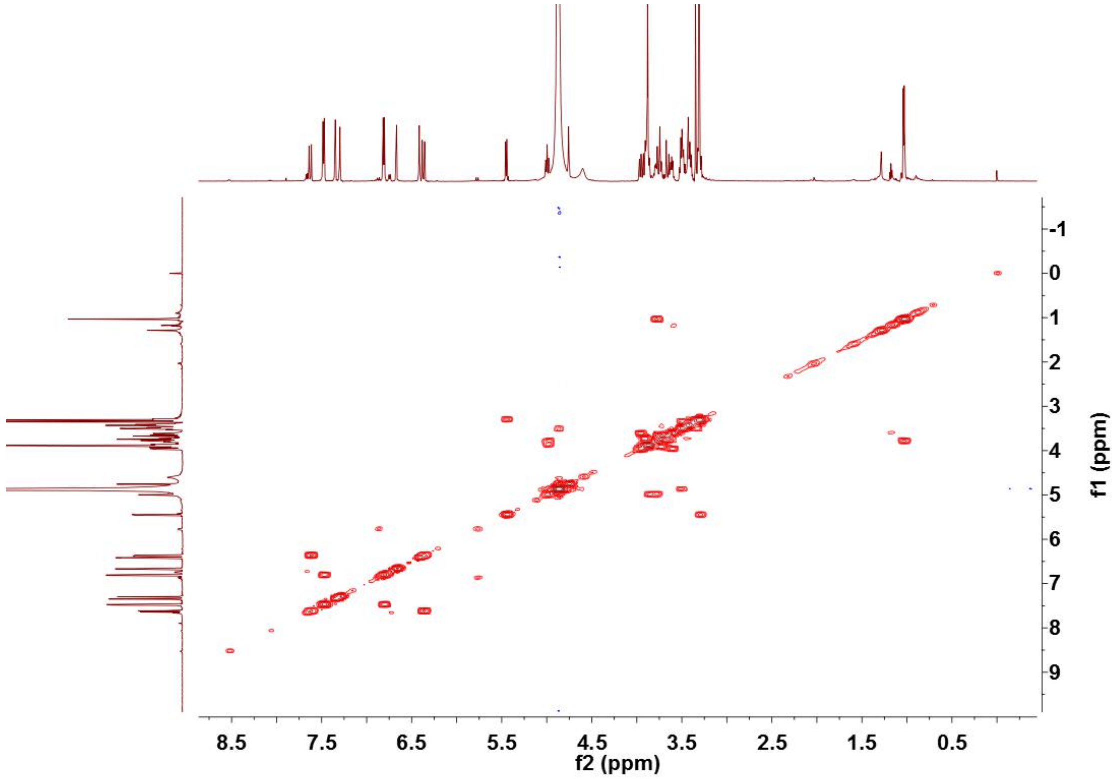

Figure S3. ^{1}H-^{1}H COSY spectrum of **1** in methanol-d_4

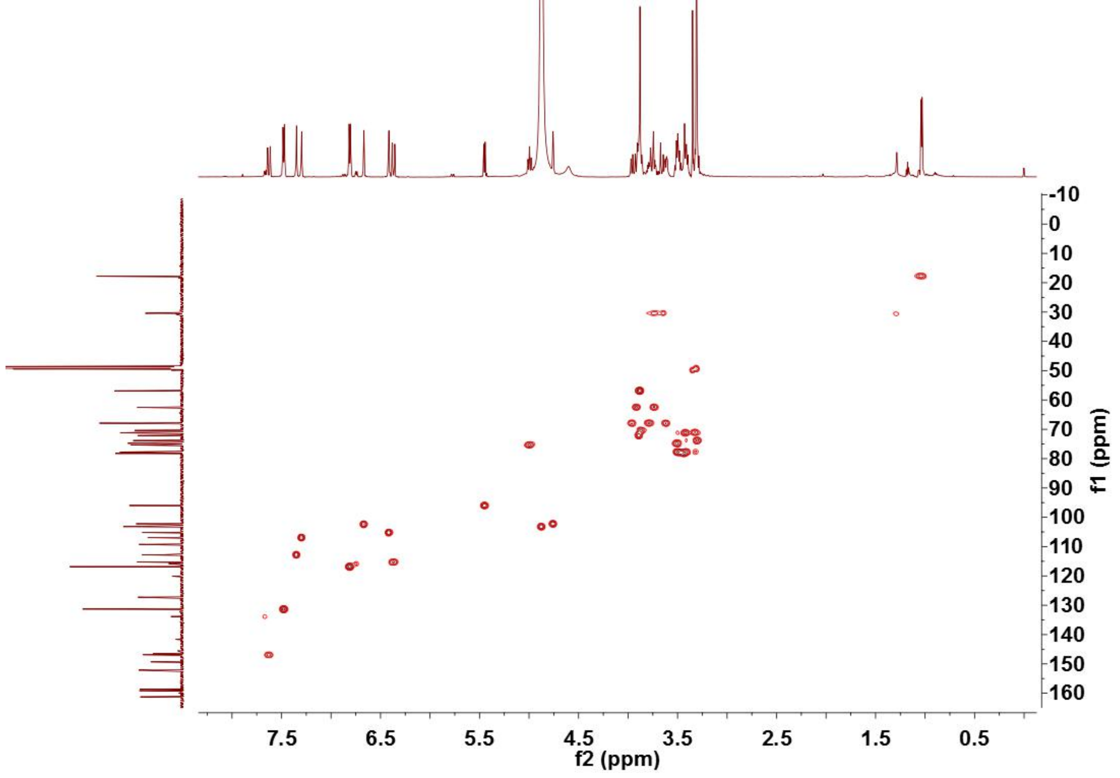

Figure S4. HSQC spectrum of **1** in methanol-d_4

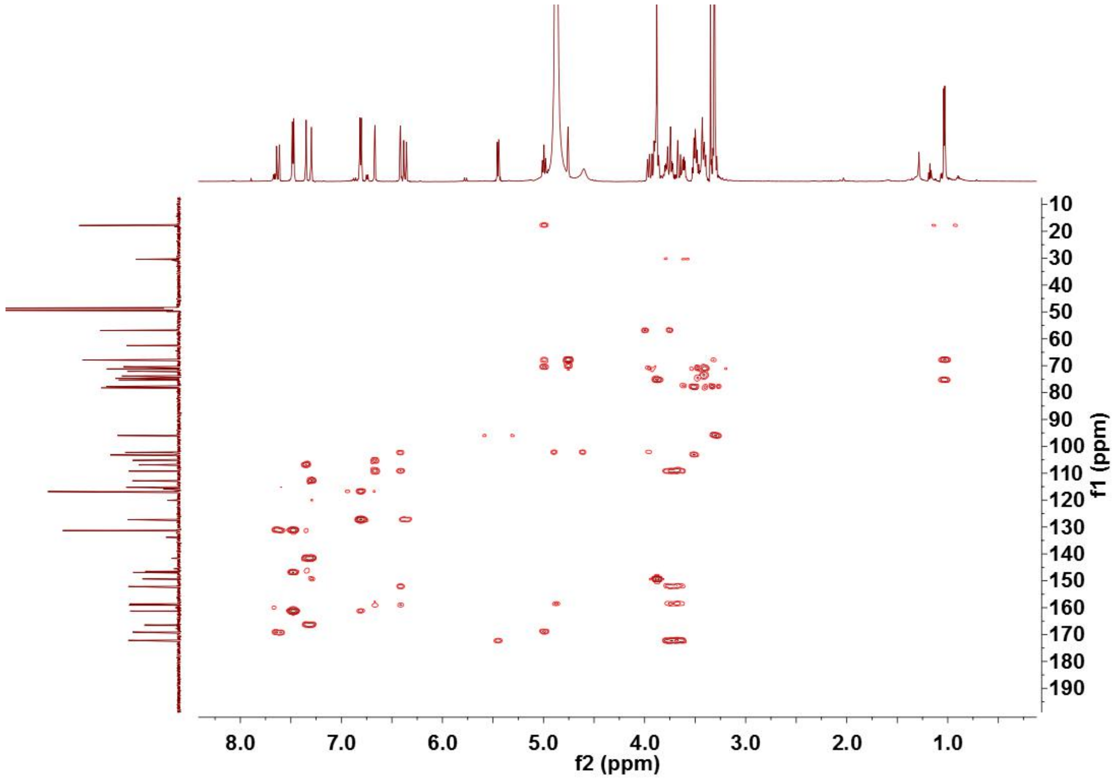

Figure S5. HMBC spectrum of **1** in methanol-d_4

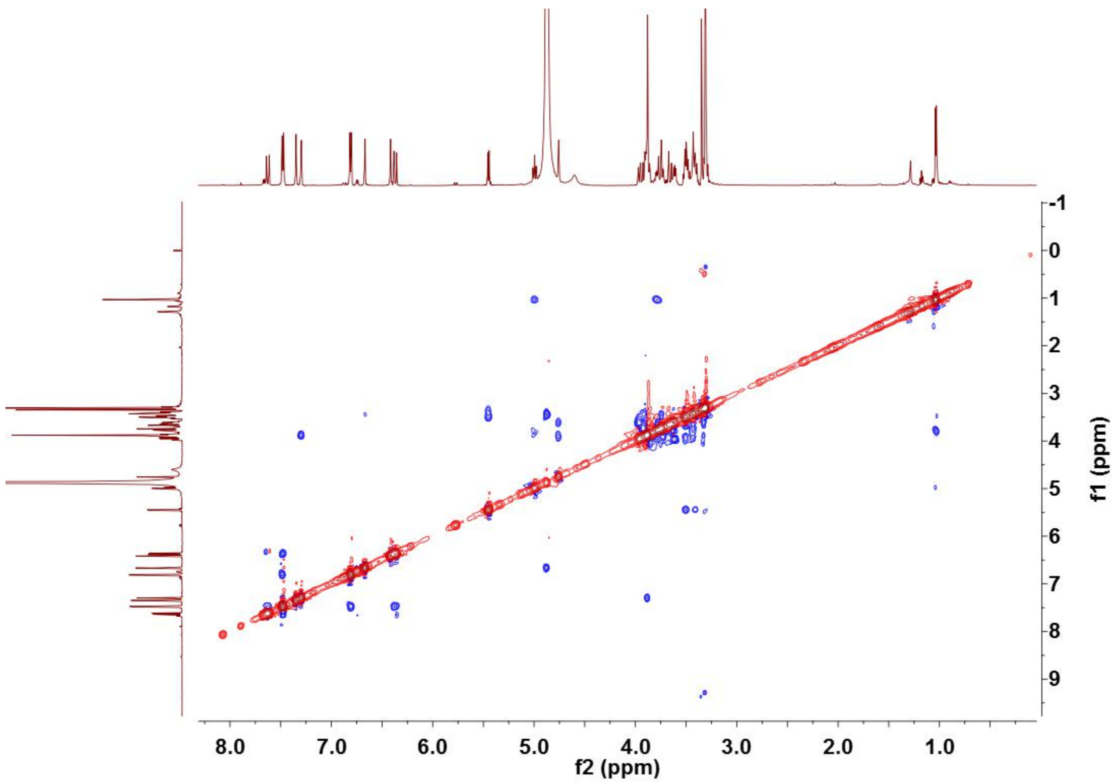

Figure S6. ROESY spectrum of **1** in methanol-d_4

Qualitative Analysis Report

**Data Filename**	180119ESIA6.d	**Sample Name**	Qg-6a
**Sample Type**	Sample	**Position**	
**Instrument Name**	Agilent G6230 TOF MS	**User Name**	KIB
**Acq Method**	ESI.m	**Acquired Time**	1/19/2018 10:53:59 AM
**IRM Calibration Status**	Success	**DA Method**	ESI.m
**Comment**			

Sample Group **Info.**
Acquisition SW 6200 series TOF/6500 series
Version Q-TOF B.05.01 (B5125.2)

User Spectra

Fragmentor Voltage	Collision Energy	Ionization Mode
200	0	ESI

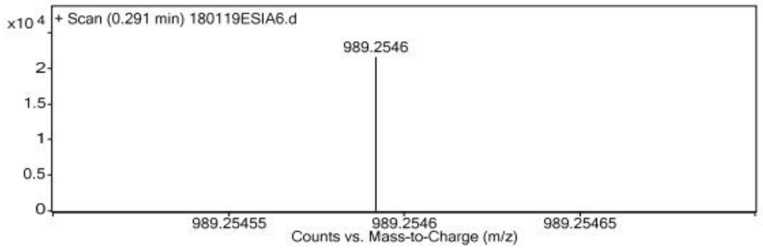

Peak List

m/z	z	Abund	Formula	Ion
119.0611	1	5677.25		
123.0922	1	14247.06		
212.119	1	68231		
213.1217	1	8095.68		
368.4261	1	8469.51		
437.2303	1	8291.43		
495.1488	1	11115.65		
549.1225	1	6741		
989.2546	1	21570.55	C43 H50 Na O25	M+
990.2571	1	8602.18	C43 H50 Na O25	M+

Formula Calculator Element Limits

Element	Min	Max
C	0	200
H	0	400
O	21	29
Na	1	1

Formula Calculator Results

Formula	CalculatedMass	Mz	Diff.(mDa)	Diff. (ppm)	DBE
C43 H50 Na O25	989.2539	989.2546	-0.7	0.7	18.5

--- End Of Report ---

Figure S7. HRESIMS spectrum of **1**

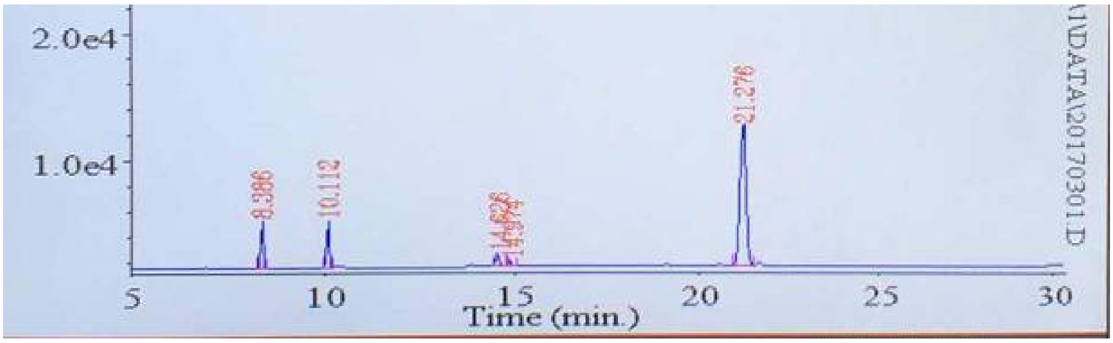

Figure S8. GC analysis of the derivative of D-glucose

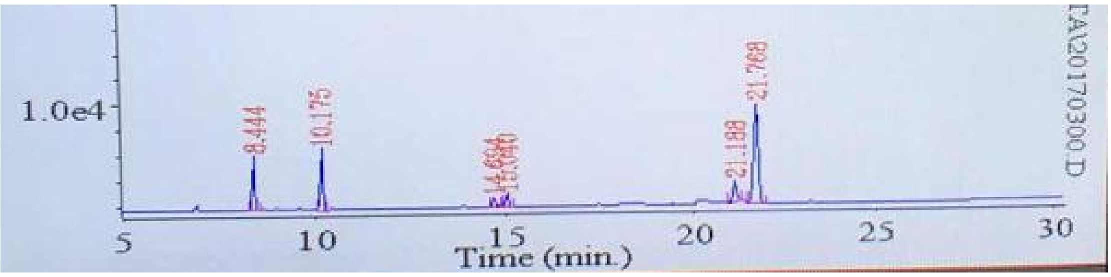

Figure S9. GC analysis of the derivative of L-glucose

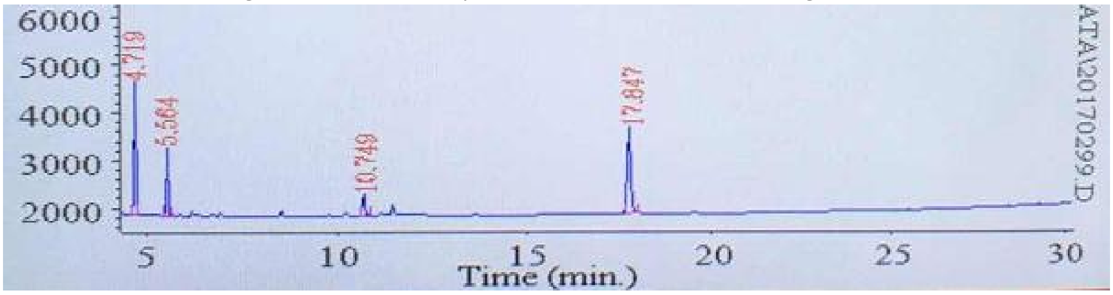

Figure S10. GC analysis of the derivative of L-rhamnose

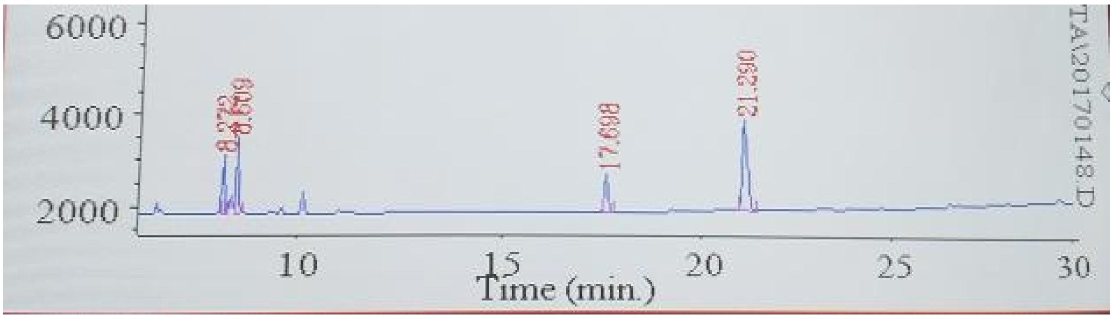

Figure S11. GC analysis of the derivative of compound **1** after hydrolysis

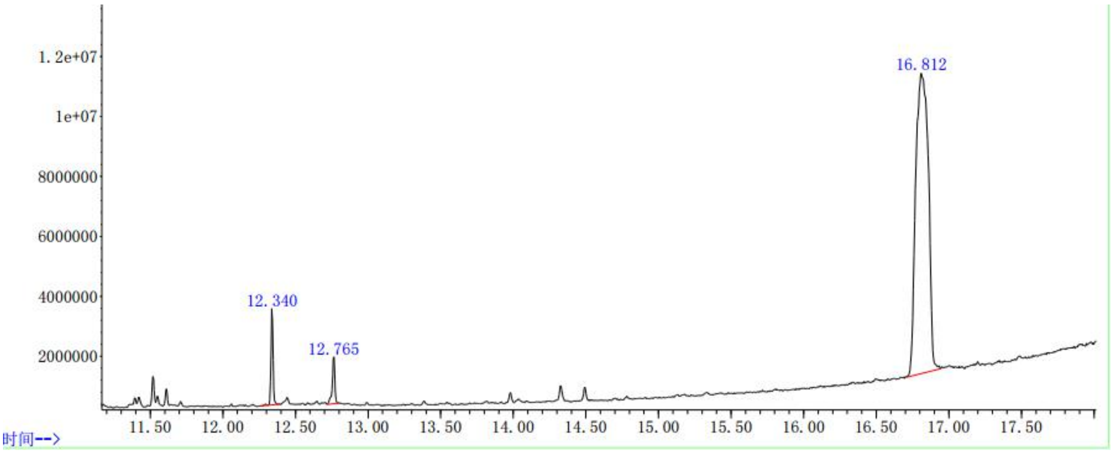

Figure S12. GC/MS analysis of the derivative of L-rhamnose

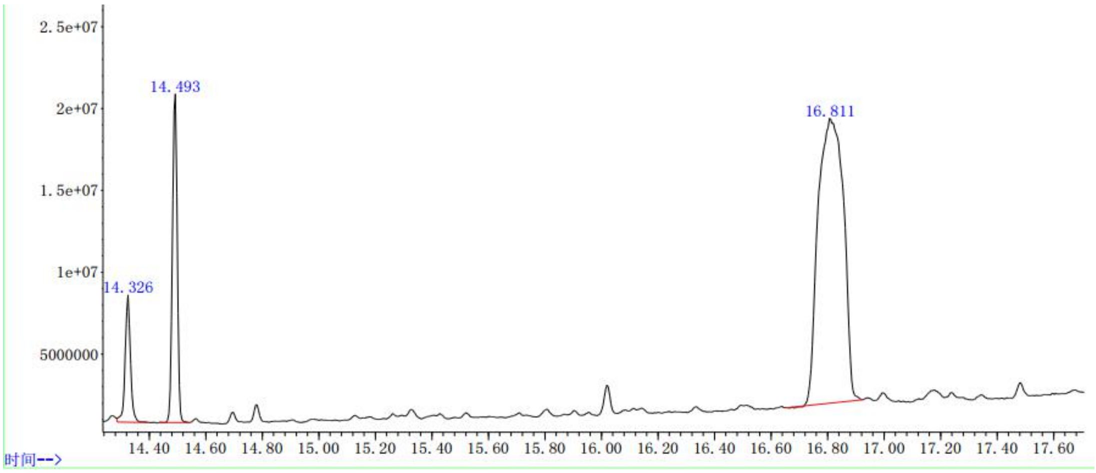

Figure S13. GC/MS analysis of the derivative of compound **1** after hydrolysis

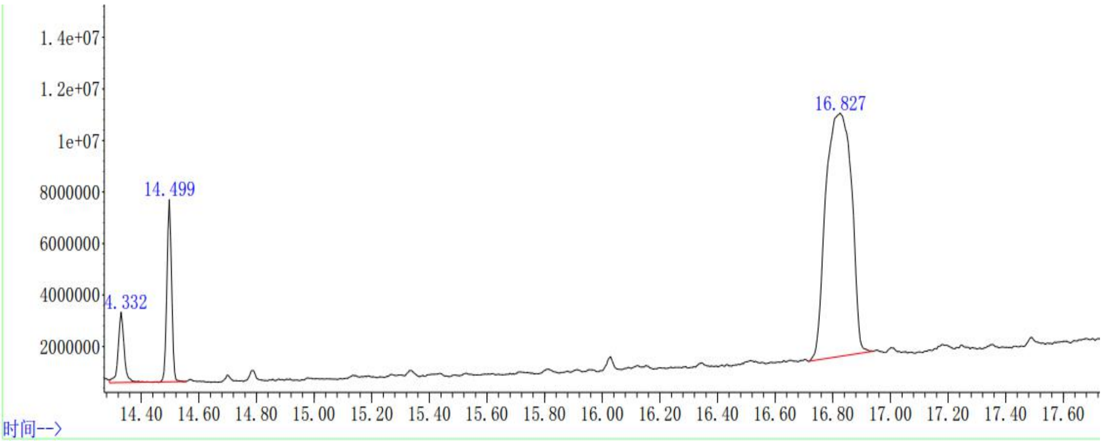

Figure S14. GC/MS analysis of the mixture of L-rhamnose with **1** after hydrolysis
